# Concomitant drugs associated with increased mortality for MDMA users reported in a drug safety surveillance database

**DOI:** 10.1038/s41598-021-85389-x

**Published:** 2021-03-16

**Authors:** Isaac V. Cohen, Tigran Makunts, Ruben Abagyan, Kelan Thomas

**Affiliations:** 1 Clinical PharMacology and Therapeutics Postdoctoral Training Program, University of California San Francisco, San Francisco, CA USA; 2 Skaggs School of Pharmacy and Pharmaceutical Sciences, University of California San Diego, La Jolla, CA USA; 3 Oak Ridge Institute of Science and Education, Clinical Pharmacology and Machine Learning Fellowship At the Center for Drug Evaluation and Research, United States Food and Drug Administration, Silver Spring, MD USA; 4 College of Pharmacy, Touro University California, Vallejo, CA USA

**Keywords:** Psychiatric disorders, Post-traumatic stress disorder, Drug development, Outcomes research, Epidemiology, Clinical pharmacology, Drug safety, Pharmaceutics, Toxicology

## Abstract

3,4-Methylenedioxymethamphetamine (MDMA) is currently being evaluated by the Food and Drug Administration (FDA) for the treatment of post-traumatic stress disorder (PTSD). If MDMA is FDA-approved it will be important to understand what medications may pose a risk of drug–drug interactions. The goal of this study was to evaluate the risks due to MDMA ingestion alone or in combination with other common medications and drugs of abuse using the FDA drug safety surveillance data. To date, nearly one thousand reports of MDMA use have been reported to the FDA. The majority of these reports include covariates such as co-ingested substances and demographic parameters. Univariate and multivariate logistic regression was employed to uncover the contributing factors to the reported risk of death among MDMA users. Several drug classes (MDMA metabolites or analogs, anesthetics, muscle relaxants, amphetamines and stimulants, benzodiazepines, ethanol, opioids), four antidepressants (bupropion, sertraline, venlafaxine and citalopram) and olanzapine demonstrated increased odds ratios for the reported risk of death. Future drug–drug interaction clinical trials should evaluate if any of the other drug–drug interactions described in our results actually pose a risk of morbidity or mortality in controlled medical settings.

## Introduction

3,4-Methylenedioxymethamphetamine (MDMA) is currently being evaluated by the Food and Drug Administration (FDA) for the treatment of posttraumatic stress disorder (PTSD). During the past two decades, “ecstasy” was illegally distributed and is purported to contain MDMA, but because the market is unregulated this “ecstasy” may actually contain adulterants or no MDMA at all^1^. In 2018, it was estimated that around 20.5 million people in the world aged 15–64 years old had used “ecstasy” during the previous year^2^. MDMA induces the synaptic release of serotonin, norepinephrine and dopamine, which at therapeutic doses of 75–125 mg in clinical trials may cause sympathomimetic adverse drug reactions such as heart palpitations, restless legs, bruxism, sweating, dry mouth and lack of appetite^3^.

Researchers in the UK and Australia have attempted to quantify ecstasy-related death rates during the past few decades from databases of coroner reports. In a UK study of ecstasy-related mortality for people aged 16–59 years old, there were 605 deaths between 1997–2007^4^. Using crime survey data they estimated that the average UK ecstasy death rate between 2001–2007 was 1.75 per 100,000 users when used as the only drug, but with coadministration of other drugs the rate was substantially higher at 10.89 per 100,000 users^4^. In an Australian study of ecstasy-related deaths for people aged 15–64 year old, there were 392 deaths between 2000–2018; during 2001–2007 the ecstasy death rate ranged between 0.05 to 0.25 per 100,000 residents^5^. There were toxicology results available for 342 deaths, and researchers also reported that concomitant administration of ecstasy with other drugs like psychostimulants (54%), alcohol (43%), opioids (30%), cannabis (25%), and benzodiazepines (23%) accounted for a greater proportion of deaths than ecstasy-only deaths (15%)^5^.

The initial goal of this study was to evaluate MDMA-related deaths from US drug safety surveillance data. Due to the paucity of reported cases of sole ingestion of MDMA, the goals of the study were revised and the analysis goal was updated with the new aim to determine the reported risks of death when additional drug classes are coingested with MDMA. Given that current PTSD treatment guidelines recommend antidepressants, we also wanted to determine if certain antidepressants are associated with an increased risk of death.

## Results

### Patient demographics and medication records

946 unique records of MDMA use were identified and isolated from the 13,773,614 records in the FAERS FDA Adverse Event Reporting System (FAERS) database. Among these records, the mean age observed was 28 years old (sd 9.7) and the mean weight was 76 kg (sd 43.3). The majority of patients with gender listed were male (70.1%).

**Figure 1:**
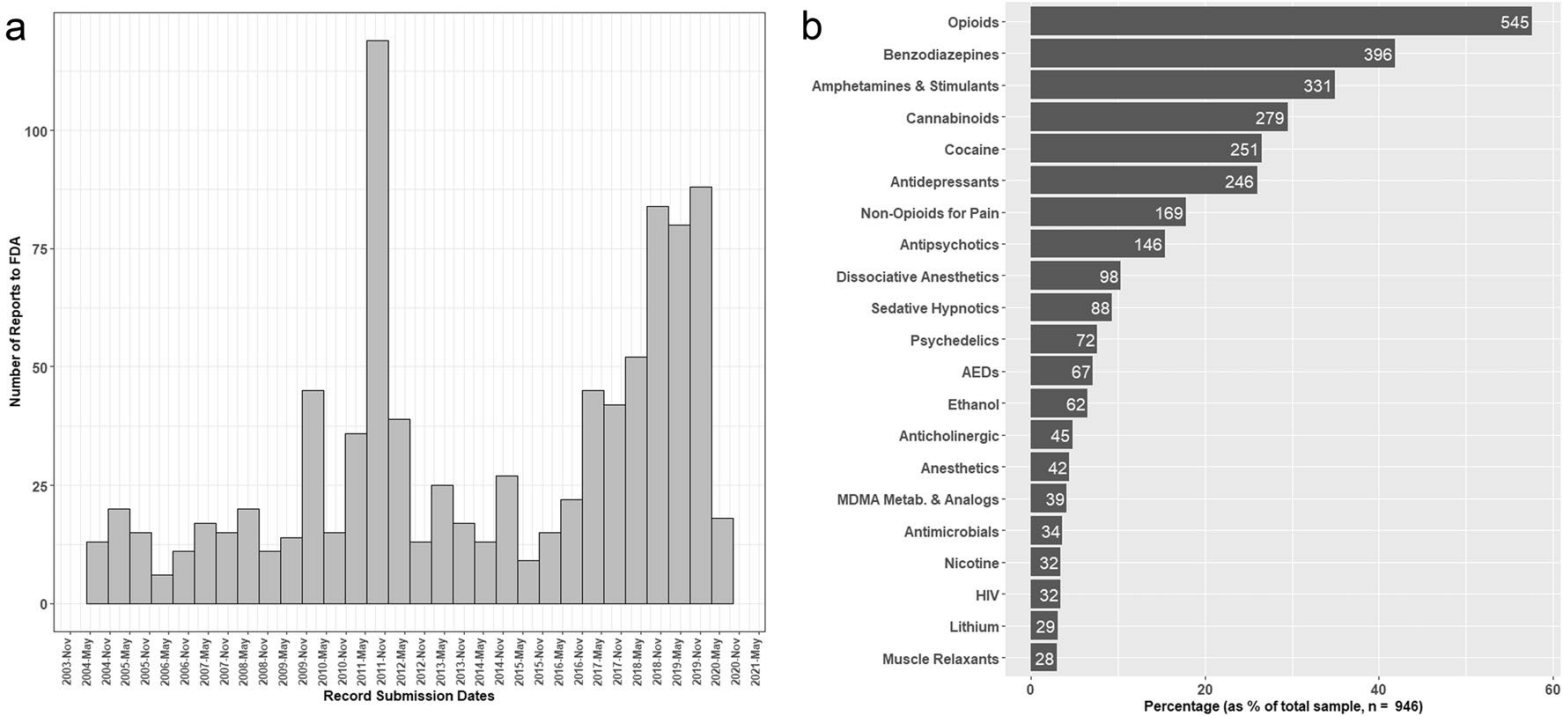
Dates of MDMA reports to FDA FAERS and frequency of reports of concomitant medications. (**a**) Histogram illustrating number of reports sent to FDA FAERS that included MDMA as a medication. Histogram bars are binned in 6-month periods. Note that reports prior to 2004 are all counted as occurred in Q1 2004. (**b**) Frequency of occurrence of one or more concomitant drugs from each individual drug class. Number of reports shown on the right side of each bar.

The majority of records of MDMA use were submitted to the FDA after 2015 (Fig. 1a). However, there was one large influx of records mostly coming from outside the United States in 2011. The cause of this large spike in reporting remains unknown. Additionally, the percentage of records containing at least one drug from each of the most common drug classes in the database is reported in Fig. 1b. The top three most common classes of concomitant drugs used in the records were opioids (58%), benzodiazepines (42%), and amphetamines/stimulants (35%) (Fig. 1b). It was found that 99.8% (944 out of 946) of the records in the dataset included at least one drug ingested in addition to MDMA. It is important to note that in the two records that listed MDMA as the only drug ingested, both records did not list death as an outcome. Additionally it should be highlighted that MDMA used by itself was very uncommon in FAERS reports, speaking to the rarity of severe adverse reactions when taken alone.

### Association of concurrent medications with all-cause mortality: univariate analysis

**Figure 2:**
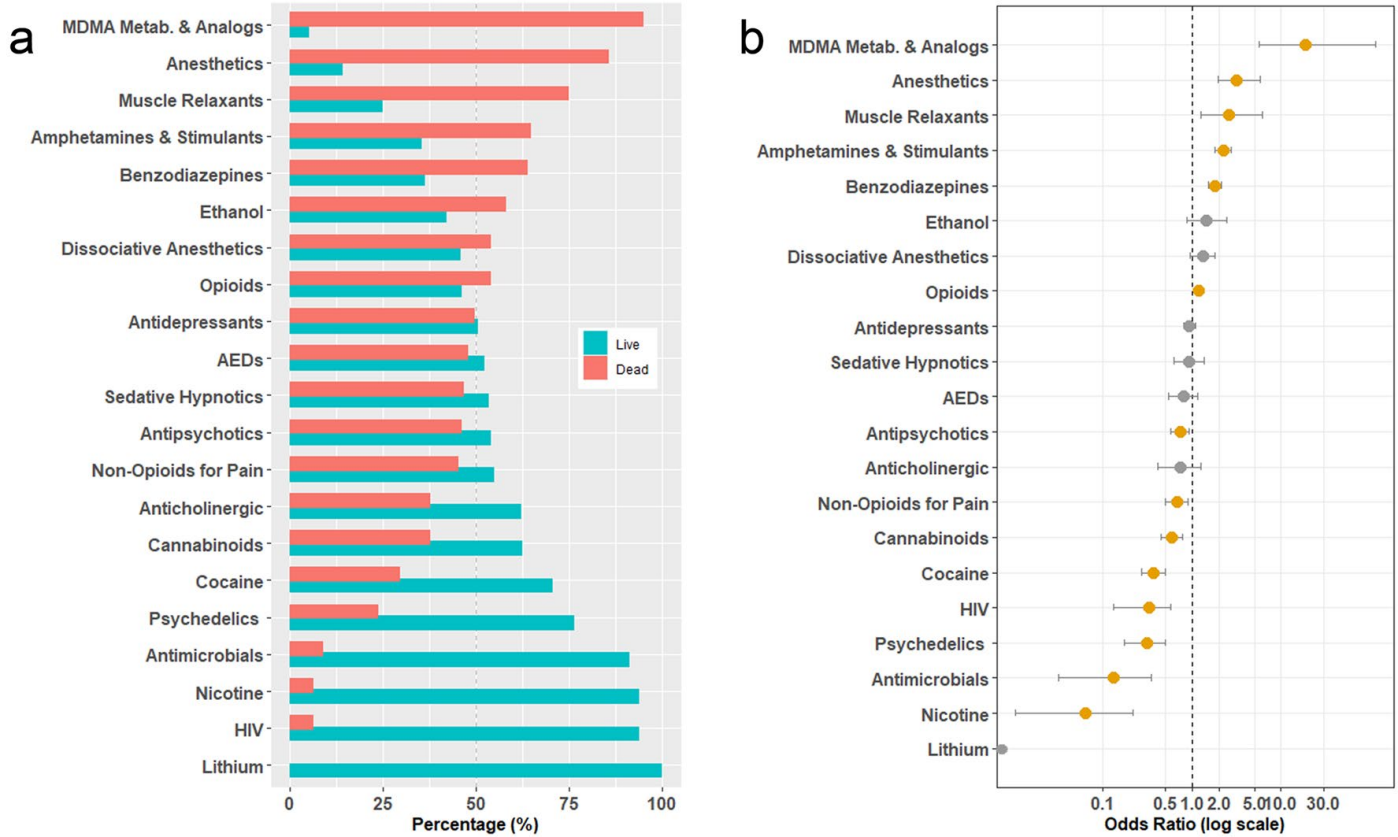
Univariate analysis of impact of concomitant drug use on odds of death in MDMA records. (**a**) Percent of records that included a given drug class that listed death as outcome shown in red. Percent of records that included a given drug class that did not list death as an outcome or adverse event shown in blue. Ordered from highest odds of death (top) to lowest odds of death (bottom). (**b**) Univariate unadjusted odds ratios for reported risk of death per additional drug added on from each class. X-axis presented here in log scale. Significant findings shown here in orange and non-significant findings are denoted in grey. 95% Confidence intervals shown as whiskers (note that the Odds Ratio for Lithium is incalculable due to no occurrences of death in the study). Odds Ratios greater than one indicate an increased reported risk of death.

It was found that the records that listed “MDMA Metabolites or Analogs” had the highest proportion of deaths in the database of all the drug classes analyzed. It was observed that 37 of the 39 (95%) records of MDMA Metabolite or Analog coingestion resulted in death. The frequencies of death among each drug class is illustrated in Fig. 2a. Figure 2b summaries the result of the univariate logistic regression modeling for the number of members of each drug class as a predictor of mortality. It was found that the number of “MDMA Metabolites or Analogs” (Odds Ratio 1.20, 95% CI [1.08–1.34]), anesthetics (3.14 [1.94–5.84]), muscle relaxants (2.62 [1.24–6.21], and amphetamines (2.23 [1.82–2.76] were the greatest predictors of mortality (Table S1 and Fig. 2b). Additionally, it was found that coingestion of lithium, nicotine, antimicrobials, HIV medications, and psychedelics were observed to have a decreased reported risk of mortality (Supplementary Table S1 and Fig. 2b). It is important to note that 100% of patients receiving lithium survived; due to no instances of death the odds ratio was not able to be calculated. Surprisingly, cocaine was observed to decrease mortality in this dataset. A full list of all the drugs included in each class is made available in Supplementary Table S2.

### Association of concurrent medications with all-cause mortality: multivariate analysis

**Figure 3:**
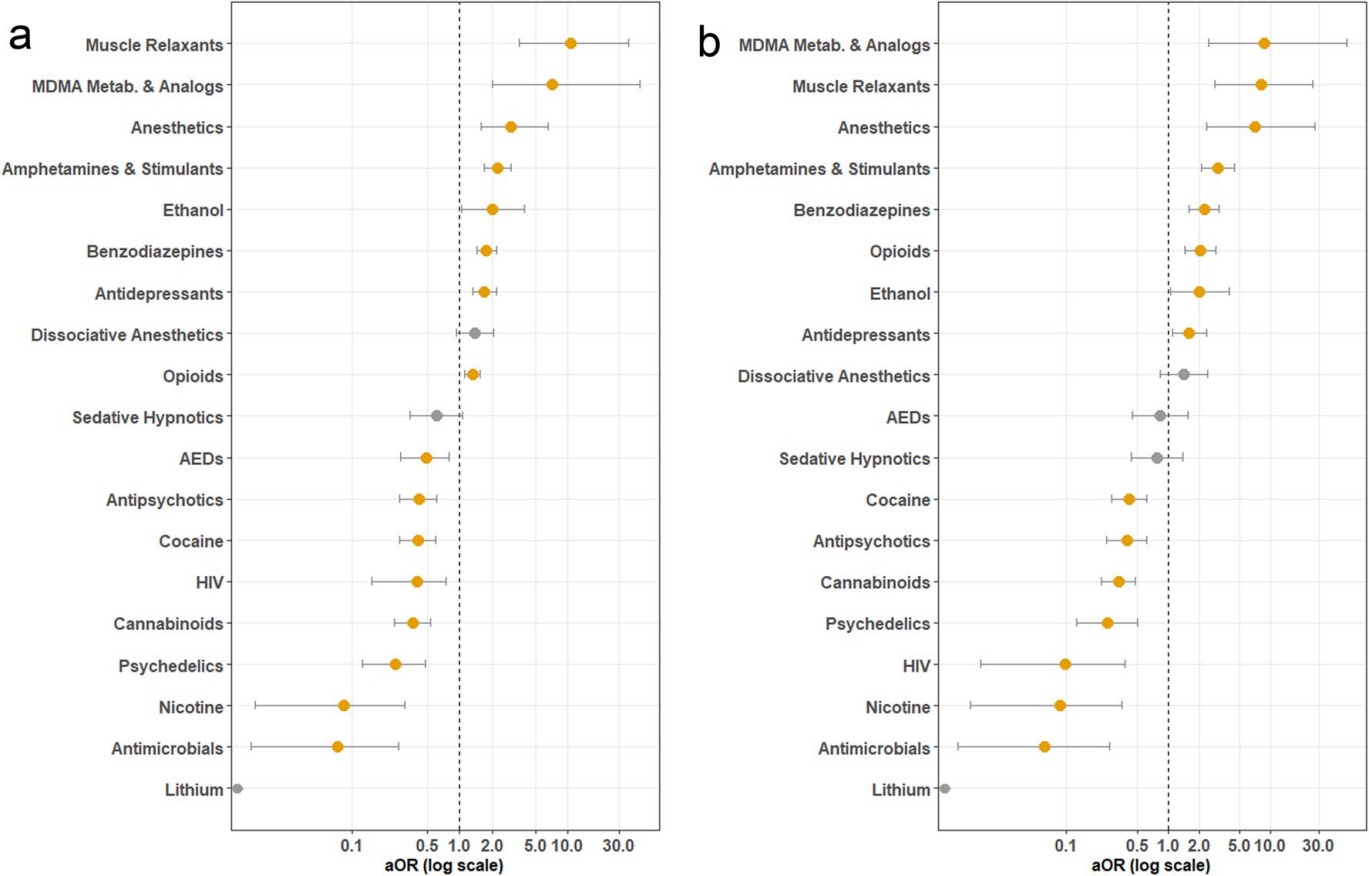
Multivariate analysis of concomitant drug use impact on odds of death in MDMA records. Multivariate adjusted odds ratios (aOR) for the reported risk of death shown for records with concomitant use of each drug class. X-axis presented here in log scale. Significant findings shown here in orange and non-significant findings are denoted in grey. 95% Confidence intervals shown as whiskers (note that the aOR for Lithium is incalculable due to no occurrences of death in the study). aORs greater than 1 indicate an increased reported risk of death. aORs less than 1 indicate a decreased reported risk of death. (**a**) The number of concomitant drugs in each class as a predictor of death. Note that only drug classes that were selected by stepwise regression are shown here. (**b**) Each drug class as a binary predictor of death. Note that only drug classes that were selected previously for panel (**a**) are shown here in panel (**b**).

In order to investigate how concurrent medications affect the rate of mortality of MDMA users, a multivariate model containing the drug classes previously discussed was built. Stepwise logistic regression was employed to eliminate any non-contributory predictors from the final model. The stepwise regression process led to an improvement of the AIC from 956.87 to 952.88 after removal of anticholinergics and non-opioid pain medications as predictors. The final model presented in Fig. 3a uses the number of concomitant drugs in each class as a predictor of the death outcome (aOR values and Confidence Intervals Reported in Supplementary Table S3).

To perform a sensitivity analysis, a duplicate model was retrained using the same database but with each drug class coded as a binary (coded as either ingested or not, instead of being coded as the number of drugs in each class). The results of this model as presented in Fig. 3b (aOR values and Confidence Intervals Reported in Supplementary Table S4). It is noteworthy that even after controlling for ingestion of other drug classes, most of the effects observed in the univariate model (Fig. 2b) were preserved.

Namely, opioids, antidepressants, benzodiazepines, amphetamines and stimulants, anesthetics, ethanol, MDMA Metabolites or Analogs, and muscle relaxants were all observed to be significant predictors of death. Of note, coingestion of some recreational substances including cannabinoids, nicotine, and psychedelics were all observed to be predictive of a decreased reported risk of mortality. Additionally, it should be highlighted that although antidepressant use was not a significant predictor of death in the univariate analysis, antidepressant use significantly increased the reported risk of the outcome of death in both multivariate analyses.

### Variable associations of specific antidepressants and antipsychotics with all-cause mortality: univariate analysis

**Figure 4:**
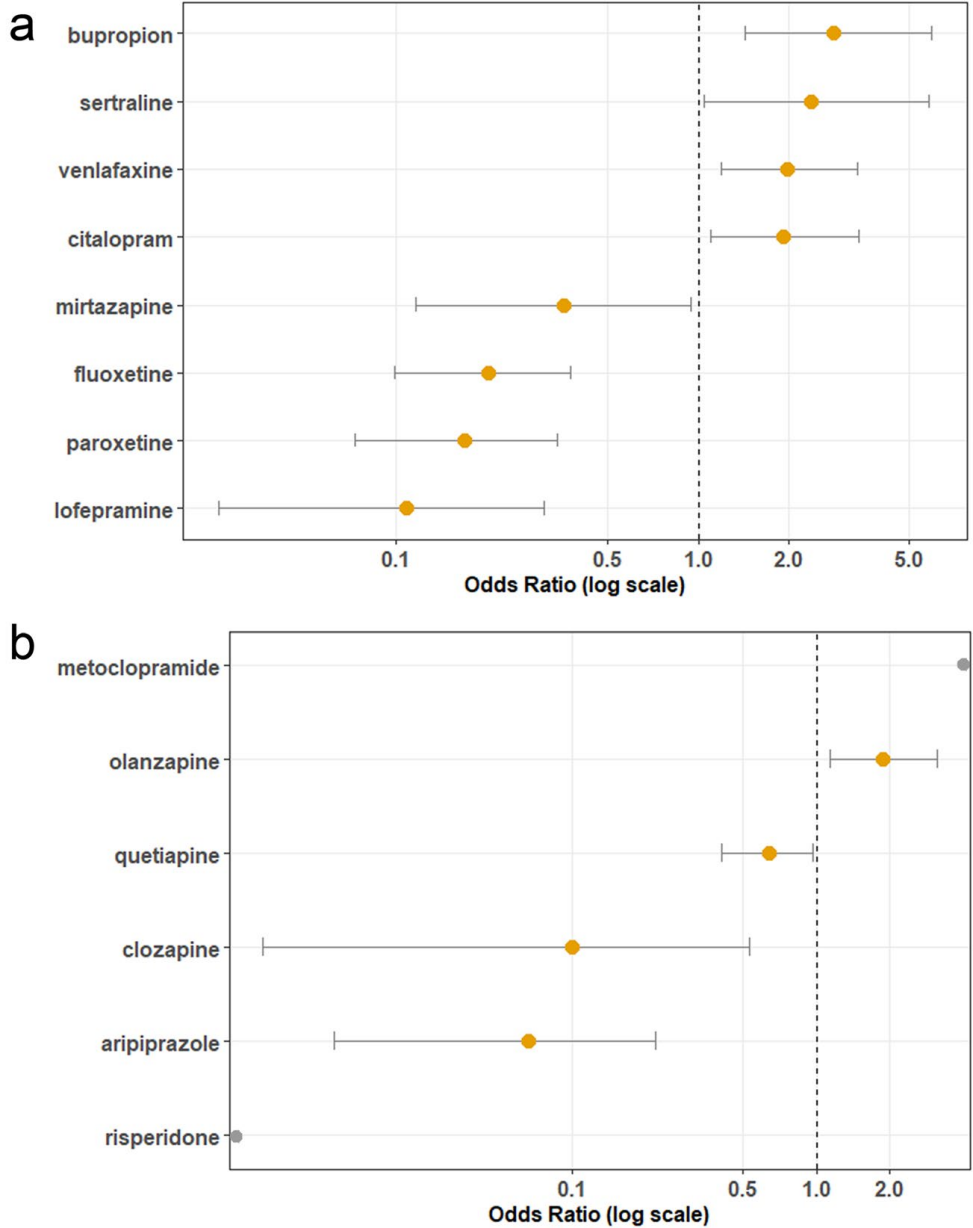
Univariate analysis of impact of common antidepressants and antipsychotics on odds of death in MDMA records. Univariate unadjusted odds ratios for the reported risk of death shown for records with concomitant use of each drug. X-axis presented here in log scale. Significant findings shown here in orange and non-significant findings are denoted in grey. 95% Confidence intervals shown as whiskers. Odds Ratios greater than 1 indicate an increased reported risk of death. (**a**) Odds of death shown for antidepressants with greater than 10 records in the dataset. (**b**) Odds of death shown for antipsychotics with greater than 10 records in the dataset (note that the odds ratio for metoclopramide is incalculable due to no occurrences of survival in the study, conversely the odds ratio for risperidone is incalculable due to no occurrences of death during the study).

Due to the antidepressant class effect on mortality and the potential likelihood of co-administration for PTSD treatment, it was decided to further investigate individual antidepressants as contributors to mortality. Only antidepressants with more than 10 reports in the dataset were included. Similar to Fig. 2b, each antidepressant was analyzed as a univariate contributor to mortality. Surprisingly, individual antidepressants were observed to both increase or decrease the reported risk of mortality (Fig. 4a, Adjusted Odds Ratios and Confidence Intervals Reported in Supplementary Table S5). Bupropion was observed to be associated with the highest reported risk of death (Odds Ratio [95% CI] 2.82 [1.43–5.96]) among the common antidepressants in the database. Venlafaxine, citalopram, and sertraline were also observed to be associated with increased reported risk of death. Conversely, paroxetine, mirtazapine, fluoxetine, and lofepramine were associated with a decreased reported risk of death.

Although antipsychotics (and metoclopramide), as a class of dopamine antagonists, were associated with a decreased reported risk of death (Figs. 2 and 3), the high variability of additional receptor activity beyond dopamine blockade in the class and occasional use for PTSD treatment lead the investigators to look deeper into the data on a per drug basis. Similar to antidepressants, it was observed that individual antipsychotics were observed to both increase or decrease reported risk of mortality (Fig. 4b, Adjusted Odds Ratios and Confidence Intervals Reported in Supplementary Table S6). Notably, in 18 out of 18 records of the dopamine antagonist metoclopramide, coingestion resulted in death (due to no cases of survival the odds ratio was not calculable). Conversely, 11 out of 11 records of risperidone coingestion did not result in mortality (due to no cases of death the odds ratio was not calculable).

### Influence of demographics on all-cause mortality: multivariate analysis

Demographic factors were also investigated as possible predictors of mortality among MDMA users. We caution that only a small fraction of the records (12.6%) included all three analyzed demographic variables: age, weight, and sex. By way of multivariate logistic regression it was found that of the three variables only age was a significant predictor of death (aOR 1.1 [95% CI 1.06–1.18], which means a 6–18% increased reported risk of death per year). In the analysis there was a non-significant trend (p = 0.06) towards low body weight contributing to an increased reported risk of death. It is important to note that the majority of our dataset did not include a full demographic description.

## Discussion

Eight drug classes (MDMA metabolites or analogs, anesthetics, muscle relaxants, amphetamines and stimulants, benzodiazepines, ethanol, opioids, antidepressants) demonstrated increased, but variable, reported risks of death when combined with MDMA. These pharmacodynamic interactions can be related to two broad pharmacologic mechanisms: (1) further enhancement of inhibitory neurotransmission or (2) further enhancement of monoamine neurotransmitter synaptic concentrations. Pharmacokinetic drug interactions are also a distinctly possible contributor, which would depend on the metabolic profile of each individual drug combination. Medications from these drug classes are already known to be dangerous in high doses, so without prospective drug–drug interaction clinical studies it is difficult to determine exactly how each co-administration with MDMA may have contributed to death. For example in one drug–drug interaction study of healthy volunteers (n = 16), methylphenidate co-administered with MDMA demonstrated a 14 bpm greater increase in heart rate than MDMA alone, so it is reasonable to hypothesize that tachycardia may have contributed to an increased MDMA mortality with stimulants^6^.

Four antidepressants (bupropion, citalopram, sertraline, and venlafaxine) demonstrated greater odds of death when combined with MDMA and should be evaluated for potential drug–drug interactions with MDMA, especially since, with the exception of bupropion, they all are routinely prescribed for PTSD treatment. One potential explanation of an elevated risk could be related to pharmacokinetics since MDMA is primarily metabolized by the CYP2D6 enzyme, while these four antidepressants are CYP2D6 inhibitors that may increase MDMA exposure. In a study of healthy volunteers (n = 16), bupropion co-administered with MDMA increased MDMA maximum concentration (Cmax) by 15% and increased area under the curve (AUC 0–24 h) by 30%^7^. Interestingly, despite this increased MDMA exposure, bupropion co-administration was actually found to attenuate the MDMA-induced increase in heart rate (HR), which at its peak effect was roughly 13 bpm lower than the average change in HR from the MDMA-only crossover arm^7^. Bupropion co-administration demonstrated no significant difference in blood pressure or temperature changes compared to the MDMA-only arm^7^. Citalopram co-administration has also been shown to attenuate the MDMA-induced increase in heart rate, along with blood pressure changes, which were on average lower by 9 bpm for HR, 9 mmHg for SBP, and 5 mmHg for DBP than the MDMA only crossover arm^8^. These antidepressant co-administration studies did not evaluate the impact of CYP2D6 metabolizer status, but another study of CYP2D6 pharmacogenomics demonstrated that poor metabolizers had 19% higher mean Cmax and 25% higher mean AUC_0–6 h_ than extensive metabolizers with only *1 or *2 alleles, which corresponded to greater elevations in peak SBP by roughly 10 mmHg^9^. The decreased reported risk of death associated with the two strongest CYP2D6 inhibitor antidepressants, fluoxetine and paroxetine, currently recommended by 2017 VA/DoD PTSD guidelines to treat PTSD casts doubt on the hypothesis that MDMA co-administration with a CYP2D6 inhibitor is responsible for the increased risk of death with these four antidepressants. When fluoxetine and paroxetine are co-administered with MDMA, they also decrease subjective psychological effects and cardiac vital signs compared to MDMA alone, despite increased MDMA Cmax and AUC exposure^10–12^. Therefore the past clinical evidence consistently demonstrates that MDMA co-administration with antidepressants will attenuate MDMA effects, so increased MDMA exposure via CYP2D6 inhibition does not offer a compelling explanation for increased risk of death in our sample. Another class of antidepressants that is known to have its own unique cardiotoxicity risk is the tricyclic antidepressants, but there were insufficient cases for analysis. However, the most dangerous antidepressant combination, which would be contraindicated with MDMA-assisted therapy due to increased serotonin toxicity risk, would be concomitant monoamine oxidase inhibitor antidepressants (MAOI’s).

Another important consideration for these antidepressant drug–drug interactions is that MDMA is also an inhibitor of CYP2D6 (resulting in autoinhibition), while all four antidepressants are minor CYP2D6 substrates, so these pharmacokinetic interactions could also potentially increase antidepressant exposure and subsequent changes in pharmacodynamic effects. In the previously mentioned study of healthy volunteers (n = 16) where bupropion was co-administered with MDMA, CYP2D6 inhibition increased bupropion Cmax by 18% and AUC_0–24 h_ by 27%. The consequences of increased bupropion exposure are well documented to have a dose-related risk of seizures^7^. Citalopram and sertraline both have warnings for QT prolongation and arrhythmia risk correlated with serum concentration, and venlafaxine has dose-related risk of hypertension. Therefore, these individual antidepressant drug–drug interactions may also be evaluated in future clinical trials. Until there is evidence from MDMA-assisted therapy drug–drug interaction clinical trials, we can hypothesize that rare and serious antidepressant adverse drug reactions such as seizures with bupropion, arrhythmias with citalopram and sertraline, or hypertension with venlafaxine may hypothetically contribute to an increased mortality risk when co-administered with MDMA in uncontrolled settings.

While some clinicians may prescribe antipsychotics to treat severe refractory cases of PTSD, the 2017 VA/DoD (Veterans Affairs/Department of Defense) PTSD guidelines suggest against this practice based on weak evidence of efficacy and the known adverse effect profile of this class^13^. Therefore, it may be prudent to avoid olanzapine co-administration with MDMA since it was associated with an increased risk of mortality with MDMA. Another dopamine antagonist, metoclopramide, had MDMA-related death as an outcome in 11 out of 11 reports with no occurrences of survival. A potential explanation for this risk is that metoclopramide has an FDA warning for its proarrhythmic effects. Metoclopramide is also a minor CYP2D6 substrate, so the addition of a CYP2D6 inhibitor like MDMA with its own cardiac effects could hypothetically increase the risk of sudden cardiac death in combination.

Overall, it has been demonstrated that MDMA may interact with common recreational drugs (including as MDMA metabolites or analogs, muscle relaxants, amphetamines and stimulants, benzodiazepines, ethanol, opioids) and commonly used medications for PTSD pharmacotherapy. Since patients with PTSD may be treated with MDMA-assisted therapy in the future it is important to characterize differences in the risk of death based on co-ingested substances. Additionally, patients undergoing MDMA-assisted therapy who are also prescribed concomitant psychotropics considered higher risk of death from our results should be closely monitored.

## Conclusion

Eight drug classes (opioids, antidepressants, benzodiazepines, amphetamines and stimulants, anesthetics, ethanol, MDMA metabolites or analogs, and muscle relaxants), four antidepressants (bupropion, citalopram, sertraline, and venlafaxine), and two dopamine antagonists (olanzapine and metoclopramide) demonstrated an increased reported risk of death based on FDA drug safety surveillance data. However, the limited evidence available from small drug–drug interaction clinical trials does not corroborate these results since bupropion and citalopram appear to have minimal safety risk and actually attenuate cardiac vital signs when coadministered with MDMA in controlled settings. Future drug–drug interaction clinical trials should evaluate if any of the other drug–drug interactions described in our results actually pose a risk of morbidity or mortality in controlled medical settings.

### Study limitations

The data presented here are *not* gathered from controlled trials. We caution readers to keep in mind the observational nature of this study and to be aware of the possibility of biases in reporting rates. Due to the voluntary nature of the FAERS/AERS reports, actual population incidences of the adverse events cannot be derived. MedWatch reporting may also be biased by newsworthiness and legal variables. The safety surveillance data misses comprehensive medical records and medication history, limiting the scope of the analysis. As with any association study, causality may not be derived from association, since the cases were not uniformly evaluated for causality by clinical specialists. In addition to missing dosing information for MDMA, the purity and dose of recreational MDMA is also not listed in the FAERS database. Recreational MDMA, or “ecstasy”, may contain no MDMA at all or may contain unknown amounts of adulterants, including but not limited to MDMA metabolites, MDMA analogues, psychedelics, amphetamines, dissociative anesthetics. The effects of these adulterants were not able to be directly accounted for in the study. Additionally, there are only two cases of MDMA as the only substance ingested in the database, so a baseline risk of death due to MDMA was not able to be established. Further, note that the aORs presented here represent only reports submitted to the database and are not directly generalizable to a specific clinical population.

Nonetheless, the postmarketing surveillance data analysis of over 900 reports provides substantial evidence and can be used to identify safety signals that have not been investigated in early phase studies or that might have gone unnoticed in smaller scale studies. Additionally, our study examines drug combinations not likely to be seen in prospective clinical studies of MDMA due to inclusion of recreational substances in our dataset.

### Generalizability of results

These reports are not from controlled trials, the MDMA doses were unknown, and there was no analytical confirmation of MDMA in systemic circulation, so these results may not be fully generalizable to MDMA-related-new drug applications entities for FDA approval.

## Methods

### FDA adverse event reporting system

The study examined over thirteen million adverse event (AE) reports available from the United States Food and Drug Administration Adverse Event Reporting System (FAERS) and its predecessor, the Adverse Event Reporting System (AERS). At the time of the study the FAERS/AERS set contained reports from years 2000–2020, all available online: https://www.fda.gov/drugs/questions-and-answers-fdas-adverse-event-reporting-system-faers/fda-adverse-event-reporting-system-faers-latest-quarterly-data-files.

### Data preparation

FAERS/AERS reports are collected through voluntary reporting (and mandatory reported for specific reporting entities such as pharmaceutical manufactures) to the FDA through the MedWatch system^14^ and stored in quarterly format data subsets with their respective parameters (age, sex, drug, AE etc.), and common case identifiers. FAERS data format changes periodically, requiring each quarterly set to be individually downloaded and standardized^15–19^. The final full data set from the FDA contained 13,773,614 reports. Since the FAERS/AERS data set has reports from all over the world with their respective brand or generic names, twelve unique terms were recognized and translated into a single generic name for MDMA.

### Cohort selection and data cleaning

946 reports of MDMA ingestion were identified and used to form the study cohort for the analysis. A histogram of the dates of these 946 reports is shown in Fig. 1a. Additionally, a summary of the demographics of the study cohort is presented in the Results section. RStudio (Version 1.2.5033) and R (Version 3.6.3)^20^ were employed for data cleaning and logistic regression modeling. FAERS/AERS data sets include a small fraction of duplicate reports. The set was scanned for these entries with the R package “${\texttt{dplyr}}$” “${\texttt{distinct}}$” function and were removed as appropriate. During the data cleaning stage, age values to be used for the demographic analyses were limited to a range of 0 to 125 years. For the purpose of our analysis for sex, only values of “${\texttt{f}}$ ” or “${\texttt{m}}$ ” from FEARS/AERS were analyzed. The R package “${\texttt{dplyr}}$” function “${\texttt{mutate}}$” and “${\texttt{str}}\_{\texttt{detect}}$” were employed for counting the number concurrent drugs of each grouping.

### Measured outcomes

The primary outcome of interest for modeling was all-cause mortality, due to the early observation that death was the most common reported adverse event in the database. The R package “${\texttt{glm}}$” was employed for logistic regression modeling via the “${\texttt{binomial}}$” family function. Death was the outcome of interest in logistic regression modeling and was coded as a binary (“${\texttt{1}}$” if occurred in the report or “${\texttt{0}}$ ” if not). Adjusted Odds Ratio (aOR) values and 95% confidence intervals (95%CI) are reported in Supplementary Tables S3 and S4. The aOR is defined as an odds ratio that controls for multiple predictor variables in a model and allows for quantification of individual contributions of different variables to a single outcome^21^, in this case, the outcome of death. The aOR is calculated from the *regression coefficients *(*C*) estimated by multivariate logistic regression by the following equation: $aOR = e^{C}$, and is intended to account for biases and associations between variables from the sample data.

### Univariate modeling of drug classes

The following drug classes were evaluated for their association with the death outcome: "Muscle Relaxants", "Lithium", "HIV" (i.e. medications for HIV), "Nicotine", "Antimicrobials" (i.e. any antibiotic, antiviral, antifungal, or antiparasitic besides HIV related medications) "MDMA Metabolites and Analogs", "Anesthetics", "Anticholinergic", "Ethanol", "AEDs" (i.e. antiepileptic drugs), "Psychedelics", "Sedative Hypnotics", "Dissociative Anesthetics", "Antipsychotics", "Non-Opioids for Pain", table "Antidepressants", "Cocaine", "Cannabinoids", "Amphetamines and Stimulants", "Benzodiazepines", and "Opioids". Each drug class was annotated in the final database as an integer equal to the number of unique members of the class the record listed as coingested. For each drug class a univariate model was tested. The results of the univariate modeling are reported in Fig. 2b and Supplementary Table S1. A full list of the substance names that composed each group of drugs is available in Supplementary Table S2.

### Multivariate model refinement

All of the drug classes tested in the univariate model (described above) were carried forward for multivariate logistic regression modeling. Bidirectional elimination selection^22^ was employed for selection of the final predictive model of death. Each drug class was coded as an integer equal to the number of unique members of the class the record listed as coingested. The stepwise regression process led to an improvement of the Akaike information criterion (AIC) from 956.87 to 952.88 after removal of “anticholinergics” and “non-opioid pain medications” as predictors. The final model results are made available in Fig. 3a and Supplementary Table S3. Additionally, as a sensitivity analysis a second model was developed that coded each drug class as a binary predictor (i.e. record either contains a member of the drug class or not). The final model results are made available in Fig. 3b and Supplementary Table S4.

### Univariate modeling of antidepressants and antipsychotics

Antidepressants with more than 10 records in the dataset were chosen for further analysis. Using the similar methods as the previous univariate model, each drug was coded as a binary predictor of death (Fig. 4a, Supplementary Table S5). The same analysis was applied to antipsychotics (including dopamine antagonist metoclopramide) with greater than 10 records (Fig. 4b, Supplementary Table S6).

## Supplementary Information

Supplementary Information.
