# Supplementary material for: Concomitant drugs associated with increased mortality for MDMA users reported in a drug safety surveillance database
Source: Sci Rep. 2021 Mar 16;11:5997. doi: 10.1038/s41598-021-85389-x (PMC7966744; doi:10.1038/s41598-021-85389-x)
Supplement: Supplementary file 1 — Supplementary Information. [file 41598_2021_85389_MOESM1_ESM.pdf]

Concomitant drugs associated with increased mortality for MDMA users reported in a drug safety surveillance database

Isaac V. Cohen[1], Tigran Makunts[2,3], Ruben Abagyan[2*], Kelan Thomas[4]

[1] Clinical Pharmacology and Therapeutics, University of California San Francisco, San Francisco, California

[2]Skaggs School of Pharmacy and Pharmaceutical Sciences, University of California San Diego, La Jolla, California

[3]Oak Ridge Institute of Science and Education, Center for Drug Evaluation and Research, United States Food and Drug Administration, Silver Spring, Maryland

[4]College of Pharmacy, Touro University California, Vallejo, CA

*Correspondence to RA: rabagyan@health.ucsd.edu

Supplementary information

Figure S1. Frequency of most common side effects

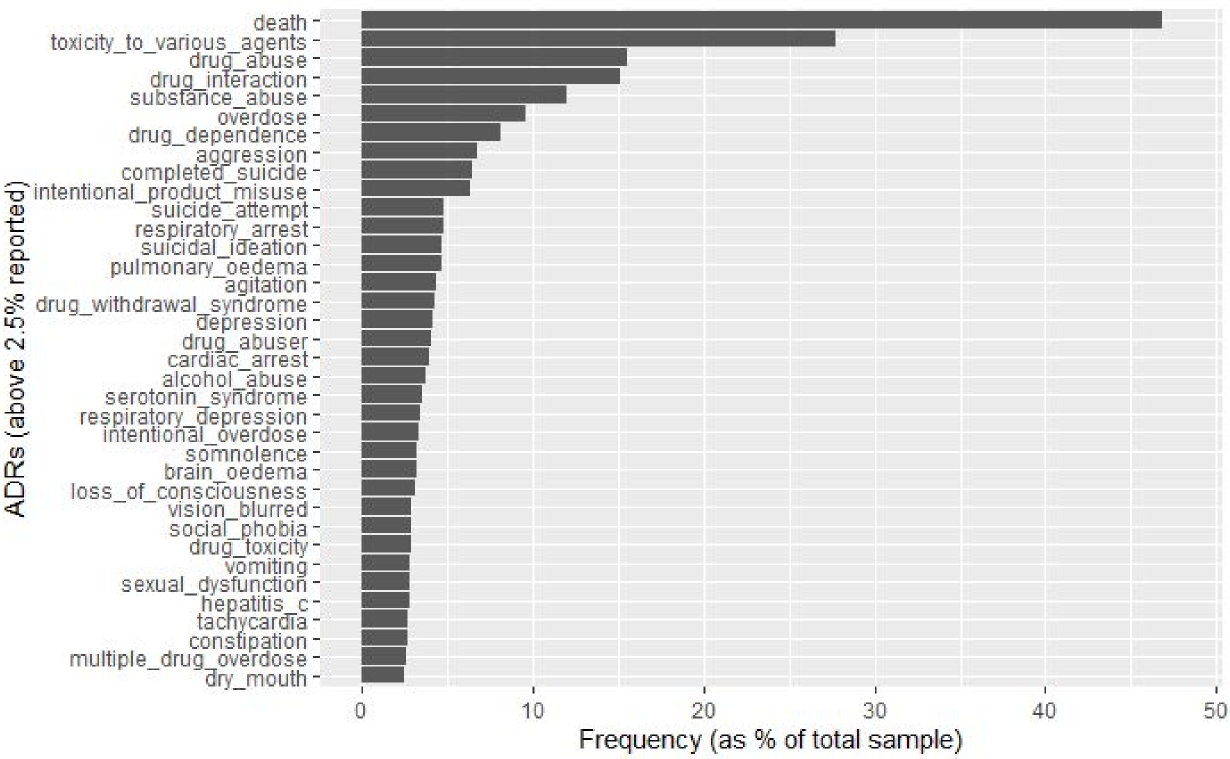

Supp. Figure 1 Legend. Frequency of occurrence of the most common side effects in the dataset (above 2.5%).

Table S1. Univariate Unadjusted Odds Ratios for Odds of Death

Class Name	OR	95%CI-Lo	95%CI-Hi
**Opioids**	1.20	1.08	1.34
**Antidepressants**	0.93	0.80	1.08
**Benzodiazepines**	1.80	1.54	2.14
**Amphetamines & Stimulants**	2.23	1.82	2.76
**Cannabinoids**	0.59	0.45	0.77
**Antipsychotics**	0.74	0.58	0.92
**Cocaine**	0.37	0.27	0.50
**Non-Opioids for Pain**	0.68	0.51	0.90
**Dissociative Anesthetics**	1.31	0.95	1.83
**Psychedelics**	0.31	0.18	0.51
**Anesthetics**	3.14	1.94	5.84
**HIV**	0.33	0.13	0.58
**AEDs**	0.81	0.54	1.15
**Sedative Hypnotics**	0.93	0.63	1.37
**Ethanol**	1.46	0.87	2.47
**Antimicrobials**	0.13	0.03	0.35
**MDMA Metab. & Analogs**	18.56	5.70	114.16
**Muscle Relaxants**	2.62	1.24	6.21
***Lithium**	NA	NA	NA
**Nicotine**	0.06	0.01	0.21
**Anticholinergic**	0.73	0.42	1.25

Supp. Table 1 Legend. Univariate unadjusted odds ratios for risk of death per additional drug added on from each class. 95% Confidence intervals reported. Odds Ratios (OR) greater than 1 indicate increased risk of death. Odds Ratios less than 1 indicate decreased risk of death. (*Note that the OR for Lithium is incalculable due to no occurrences of death in the study).

Table S2. Drugs Included in Study and their Drug Class Assignments

Drug Class	**Drug Name**
AED	pregabalin
AED	lamotrigine
AED	valproic_acid
AED	gabapentin
AED	levetiracetam
AED	topiramate
AED	briviact
AED	depamide
AED	lacosamide
AED	phenytoin
AED	carbamazepine
AED	oxcarbazepine
Amphetamines & Stimulants	methamphetamine
Amphetamines & Stimulants	amphetamine
Amphetamines & Stimulants	methylphenidate
Amphetamines & Stimulants	amfetamine
Amphetamines & Stimulants	methylenedioxyamphetamine
Amphetamines & Stimulants	amphetamines
Amphetamines & Stimulants	metamfetamine
Amphetamines & Stimulants	pseudoephedrine
Amphetamines & Stimulants	adderall
Amphetamines & Stimulants	amphetamine,dextroamphetamine
Amphetamines & Stimulants	dextroamphetamine_and_amphetamine_salts
Amphetamines & Stimulants	modafinil
Amphetamines & Stimulants	amfetamine_sulfate
Amphetamines & Stimulants	atomoxetine
Amphetamines & Stimulants	centrally_acting_sympathomimetics
Amphetamines & Stimulants	ibuprofen,pseudoephedrine
Amphetamines & Stimulants	actifed
Amphetamines & Stimulants	armodafinil
Amphetamines & Stimulants	cathinone
Amphetamines & Stimulants	cathinone_derivatives_no_pref_name
Amphetamines & Stimulants	ephedrine
Amphetamines & Stimulants	mcat
Amphetamines & Stimulants	mephedrone
Amphetamines & Stimulants	n_methylephedrone
Amphetamines & Stimulants	paramethoxymethamphetamine_no_pref_name

Amphetamines & Stimulants	ritaline
Amphetamines & Stimulants	speed
Amphetamines & Stimulants	3_fluorophenmetrazine
Amphetamines & Stimulants	4_mec
Amphetamines & Stimulants	4_methyl_n_ethyl_pentedrone
Amphetamines & Stimulants	4_methylmethcathinone
Amphetamines & Stimulants	amphetamine_no_pref_name
Amphetamines & Stimulants	amphetamine_salts_amfetamine_salts
Amphetamines & Stimulants	amphetamine_salts_no_pref_name
Amphetamines & Stimulants	crystal_methamphetamine
Amphetamines & Stimulants	epinephrine
Amphetamines & Stimulants	lisdexamfetamine
Amphetamines & Stimulants	metamfetamine_hcl
Amphetamines & Stimulants	metamphetamine
Amphetamines & Stimulants	methamphetamine_no_pref_name
Amphetamines & Stimulants	methamphetamines
Amphetamines & Stimulants	methylamphetamine
Amphetamines & Stimulants	norepinephrine
Amphetamines & Stimulants	phentermine
Amphetamines & Stimulants	phenylephrine
Amphetamines & Stimulants	stimulants
Anesthetics	epinephrine,lidocaine
Anesthetics	rocuronium
Anesthetics	anesthetics
Anesthetics	benzocaine
Anesthetics	bupivacaine
Anesthetics	local_anesthetics
Anesthetics	procaine
Anesthetics	suxamethonium
Anesthetics	vecuronium
Anticholinergic	diphenhydramine
Anticholinergic	promethazine
Anticholinergic	diphenhydramine,zinc
Anticholinergic	trihexyphenidyl
Anticholinergic	atropine
Anticholinergic	dimenhydrinate
Anticholinergic	benzatropine
Anticholinergic	acetaminophen,diphenhydramine
Anticholinergic	donormyl

Anticholinergic	donormyl_doxylamine_sodium_bromide_sodium_phosphate_dibasic_sodium_sulfate
Anticholinergic	scopolamine
Anticholinergic	tiotropium
Antidepressants	fluoxetine
Antidepressants	venlafaxine
Antidepressants	citalopram
Antidepressants	paroxetine
Antidepressants	bupropion
Antidepressants	lofepramine
Antidepressants	sertraline
Antidepressants	mirtazapine
Antidepressants	desvenlafaxine
Antidepressants	antidepressants
Antidepressants	trazodone
Antidepressants	seroxat_smith_kline_beecham
Antidepressants	escitalopram
Antidepressants	dothiepin_hcl
Antidepressants	moclobemide
Antidepressants	reboxetine
Antidepressants	dothiepin_00160401
Antidepressants	doxepin
Antidepressants	duloxetine
Antidepressants	amitriptyline
Antidepressants	antidepressant_nos
Antidepressants	desipramine
Antidepressants	dothiepin
Antidepressants	efexor
Antidepressants	nortriptylin
Antidepressants	phenelzine
Antidepressants	ratio_venlafaxine
Antidepressants	selective_serotonin_reuptake_inhibitor
Antidepressants	sertralin_01011402
Antidepressants	st_john_s_wort
Antidepressants	tricyclic_antidepressants
Antimicrobials	clarithromycin
Antimicrobials	azithromycin
Antimicrobials	clindamycin
Antimicrobials	glecaprevir,pibrentasvir
Antimicrobials	fluconazole

Antimicrobials	levamisole
Antimicrobials	sulfamethoxazole,trimethoprim
Antimicrobials	amoxicillin
Antimicrobials	cephalexin
Antimicrobials	doxycycline
Antimicrobials	pentamidine
Antimicrobials	telithromycin
Antimicrobials	aciclostad
Antimicrobials	amoxi_1a_pharma
Antimicrobials	amoxicillin,clavulanate
Antimicrobials	ceftriaxon_actavis
Antimicrobials	doxycyclin_00055701
Antimicrobials	fluconazole_in_sodium_chloride_injection_usp
Antimicrobials	fluoroquinolones
Antimicrobials	magistrale
Antimicrobials	moxifloxacin
Antimicrobials	nitrofurantoin
Antipsychotics	olanzapine
Antipsychotics	aripiprazole
Antipsychotics	quetiapine
Antipsychotics	metoclopramide
Antipsychotics	clozapine
Antipsychotics	risperidone
Antipsychotics	chlorpromazine
Antipsychotics	cyamemazine
Antipsychotics	haloperidol
Antipsychotics	propiomazine
Antipsychotics	prochlorperazine
Antipsychotics	ziprasidone
Antipsychotics	domperidone
Antipsychotics	fluphenazine
Antipsychotics	paliperidone
Antipsychotics	quetiapin
Antipsychotics	zuclopenthixol
Antipsychotics	ability_aripiprazole
Antipsychotics	amisulpride
Antipsychotics	antipsychotics
Antipsychotics	chlorprothixene
Antipsychotics	dominal

Antipsychotics	levomepromazine
Antipsychotics	methotrimeprazine
Antipsychotics	neuleptil_periciazine
Antipsychotics	pipamperone
Antipsychotics	thioridazine
Benzodiazepines	diazepam
Benzodiazepines	alprazolam
Benzodiazepines	clonazepam
Benzodiazepines	oxazepam
Benzodiazepines	temazepam
Benzodiazepines	lorazepam
Benzodiazepines	nordazepam
Benzodiazepines	midazolam
Benzodiazepines	bromazepam
Benzodiazepines	nordiazepam
Benzodiazepines	nitrazepam
Benzodiazepines	benzodiazepine_derivatives
Benzodiazepines	7_aminoflunitrazepam_flunitrazepam
Benzodiazepines	benzodiazepine
Benzodiazepines	flunitrazepam
Benzodiazepines	1_hydroxymidazolam
Benzodiazepines	benzodiazepines
Benzodiazepines	7_aminoflunitrazepam
Benzodiazepines	clotiazepam
Benzodiazepines	noctamide
Benzodiazepines	nordiazepam_no_pref_name
Benzodiazepines	prazepam
Benzodiazepines	tavor
Benzodiazepines	veratran
Benzodiazepines	benzodiazepine_related_drugs
Benzodiazepines	benzos_benzodiazepines
Benzodiazepines	chlordiazepoxide
Benzodiazepines	etizolam
Benzodiazepines	flurazepam
Benzodiazepines	normabel
Benzodiazepines	rohipnol
Benzodiazepines	temaz
Benzodiazepines	valium_00017001
Cannabinoids	cannabis

Cannabinoids	marijuana
Cannabinoids	dronabinol
Cannabinoids	tetrahydrocannabinol
Cannabinoids	8_tetrahydrocannabinol
Cannabinoids	cannabis_sativa
Cannabinoids	thc
Cannabinoids	cannabis_et_resine
Cannabinoids	thc_cannabis_sativa
Cannabinoids	thc_no_pref_name
Cannabinoids	cannabis_resin
Cannabinoids	cannabis_sativa_oil
Cannabinoids	marijuana_cannabis
Cannabinoids	marijuana_cannibas
Cannabinoids	ab_fubinaca
Cannabinoids	cannabinoids
Cannabinoids	cannabis_and_resin
Cannabinoids	cannabis_cannabis_sativa
Cannabinoids	cannabis_formulation_unknown_cannabis
Cannabinoids	cannabis_sativa_cannabis_cannabis_sativa
Cannabinoids	cannabis_sativa_subsp_indica_top
Cannabinoids	cannabis_sativa_subsp_sativa_flowering_top
Cannabinoids	cannabis_tea
Cannabinoids	delta_9_carboxy_tetrahydrocannabinol
Cannabinoids	marijuana_cannabis_sativa
Cannabinoids	marijuana_no_pref_name
Cannabinoids	marijuana_thc_hemp_hash
Cannabinoids	thc_cannabis_staiva
Cannabinoids	thc_cannabs_satva
Cannabinoids	unspecified_cannabinoids
Cannabinoids	unspecified_formulatin_of_tetrahydrocannasinol_thc
Cocaine	cocaine
Cocaine	benzoylecgonine
Cocaine	crack_cocaine
Cocaine	ecgonine
Dissociative_Anesthetics	ketamine
Dissociative_Anesthetics	norketamine
Dissociative_Anesthetics	phencyclidine
Dissociative_Anesthetics	propofol
Dissociative_Anesthetics	nitrous_oxide

Dissociative_Anesthetics	pcp
Dissociative_Anesthetics	3_methoxyphencyclidine
Dissociative_Anesthetics	methoxamine
Dissociative_Anesthetics	norketamie
Ethanol	ethanol
Ethanol	alcool
Ethanol	lager
Ethanol	alcool_modifie
HIV	ritonavir
HIV	darunavir
HIV	raltegravir
HIV	emtricitabine
HIV	emtricitabine,tenofovir
HIV	atazanavir
HIV	emtricitabine,tenofovir_disoproxil
HIV	abacavir
HIV	lamivudine
HIV	tenofovir
HIV	zidovudine
HIV	acyclovir
HIV	bms561390
HIV	didanosine
HIV	ledipasvir
HIV	nevirapine
HIV	saquinavir
HIV	tmc_114
HIV	cobicistat,elvitegravir,emtricitabine,tenofovir
HIV	cobicistat,elvitegravir,emtricitabine,tenofovir_alafenamide
HIV	cobicistat,elvitegravir,emtricitabine,tenofovir_disoproxil
HIV	dolutegravir
HIV	emtricitabine,tenofovir_alafenamide
HIV	emtricitabine_tenofovir_disoproxil_tenofovir_disoproxil_fumarate
HIV	eviplera
HIV	rilpivirine
Lithium	lithium
MDMA_metabs_analogs	mda
MDMA_metabs_analogs	tenamfetamine
MDMA_metabs_analogs	para_methoxymethamphetamine
MDMA_metabs_analogs	methylene_dioxyamphetamine

MDMA_metabs_analogs	paramethoxyamphetamine_no_pref_name
MDMA_metabs_analogs	5_6_methylenedioxy_2_aminoindane_mdai
MDMA_metabs_analogs	mdai
MDMA_metabs_analogs	methoxyamphetamine
MDMA_metabs_analogs	methylone
MDMA_metabs_analogs	n_ethylpentylone
MDMA_metabs_analogs	p_methoxyamphetamine_no_pref_name
MDMA_metabs_analogs	paramethoxymethamphetamine
MDMA_metabs_analogs	pma
MDMA_metabs_analogs	pmma
MDMA_metabs_analogs	mda_ecstasy
Muscle_Relaxant	methocarbamol
Muscle_Relaxant	cyclobenzaprine
Muscle_Relaxant	carisoprodol
Muscle_Relaxant	baclofen
Muscle_Relaxant	muscle_relaxants
Muscle_Relaxant	naprotag_flex_naproxen_carisoprodol
Muscle_Relaxant	orphenadrine
Muscle_Relaxant	tizanidine
Nicotine	nicotine
Nicotine	tobacco
Nicotine	nicotiana
Nicotine	tabac_poudre_de
Nicotine	tabacum
Nicotine	varenicline
NonOpioid_Pain	acetaminophen
NonOpioid_Pain	ibuprofen
NonOpioid_Pain	aspirin
NonOpioid_Pain	diclofenac
NonOpioid_Pain	anadin_ibuprofen
NonOpioid_Pain	naproxen
NonOpioid_Pain	paracetamol
NonOpioid_Pain	anadin_ibuprofen_no_pref_name
NonOpioid_Pain	ibuprofen_oral_suspension_ups_100_mg_5_ml_rx_alpharma_ibuprofen_o
NonOpioid_Pain	nuprofen
Opioids	methadone
Opioids	morphine
Opioids	codeine
Opioids	oxycodone

Opioids	heroin
Opioids	buprenorphine
Opioids	fentanyl
Opioids	acetaminophen,oxycodone
Opioids	tramadol
Opioids	hydromorphone
Opioids	tilidine
Opioids	hydrocodone
Opioids	dihydrocodeine
Opioids	hydrocodone,acetaminophen
Opioids	acetaminophen,codeine
Opioids	acetaminophen,hydrocodone
Opioids	substitol
Opioids	pethidine
Opioids	co_proxamol
Opioids	diamorphine
Opioids	monoacetyl_morphine
Opioids	non_pmn_buprenorphine
Opioids	opioids
Opioids	6_mam_diamorphine
Opioids	6_mam_no_pref_name
Opioids	burprenorphine
Opioids	codoliprane
Opioids	ketobemidone
Opioids	opioid_analgesic
Opioids	opioid_analgesic_nos
Opioids	prontalgine
Opioids	u_47700
Opioids	6_mam
Opioids	acetaminophen,caffeine,dihydrocodeine
Opioids	acetaminophen_butalbital_caffeine_codeine
Opioids	acetaminophen_caffeine_citrate_codeine_phosphate
Opioids	acetaminophen_caffeine_codeine_phosphate
Opioids	acetaminophen_hydrocodone_no_pref_name
Opioids	acetaminophen_with_propoxyphene_hcl
Opioids	black_tar_heroin
Opioids	buprenorphin
Opioids	butalbital_asa_caffeine_codeine
Opioids	chlorhydrate_de_tramadol

Opioids	dextropropoxyphene
Opioids	dhc
Opioids	diamorphine_hcl
Opioids	endone
Opioids	heroin_diamorphine
Opioids	heroin_diamorphine_diamorphine
Opioids	lamaline
Opioids	lamaline_acetaminophen_belladonna_leaf_caffeine_opium
Opioids	lortab_loratadine
Opioids	methadon_amidone_hcl
Opioids	methadon_hcl
Opioids	morphine_sulfate_unk
Opioids	opium_morphine
Opioids	oramorph_sr_sustained_release
Opioids	oxycontin_talbets_oxycodone_hcl
Opioids	oxymorphone
Opioids	panlor
Opioids	paregoric_liquid_usp_alpharma_paregoric
Opioids	remifentanil
Opioids	skenan
Psychedelics	lsd
Psychedelics	lysergide
Psychedelics	lysergic_acid_diethylamide
Psychedelics	psilocybine
Psychedelics	dimethyltryptamine
Psychedelics	dmt_01757201
Psychedelics	dmt_dimethyltryptamine
Psychedelics	ergot_alkaloids
Psychedelics	hallucinogenc_mushroom
Psychedelics	hallucinogenic_mushroom
Psychedelics	lsd_tartrate_de
Psychedelics	lysergic_acid_diethylamide_lsd
Psychedelics	mushroom
Psychedelics	mushroom_tea_psilocybine
Psychedelics	mushrooms
Psychedelics	other_psychostimulants_and_nootropics
Psychedelics	lsd_25
Sedative Hypnotics	zolpidem
Sedative Hypnotics	ghb

Sedative Hypnotics	gamma_hydroxybutyrate
Sedative Hypnotics	melatonin
Sedative Hypnotics	zopiclone
Sedative Hypnotics	gamma_butyrolactone
Sedative Hypnotics	oxybate_sodium
Sedative Hypnotics	clonidine
Sedative Hypnotics	phenobarbital
Sedative Hypnotics	zaleplon
Sedative Hypnotics	barbiturates_and_derivatives
Sedative Hypnotics	sleeping_pills
Sedative Hypnotics	ximovan
Sedative Hypnotics	gamma_hydroxybutyric_acid_ecstasy
Sedative Hypnotics	sodium_oxybate

Table S3. Multivariate Adjusted Odds Ratios for Odds of Death

Class Name	aOR	95%CI-Lo	95%CI-Hi
**Opioids**	1.31	1.11	1.55
**Antidepressants**	1.67	1.31	2.16
**Benzodiazepines**	1.75	1.42	2.19
**Amphetamines & Stimulants**	2.21	1.69	2.94
**Cannabinoids**	0.37	0.25	0.54
**Antipsychotics**	0.42	0.28	0.61
**Cocaine**	0.41	0.28	0.60
**Dissociative Anesthetics**	1.37	0.93	2.06
**Psychedelics**	0.25	0.12	0.48
**Anesthetics**	2.95	1.56	6.49
**HIV**	0.40	0.15	0.75
**AEDs**	0.49	0.29	0.80
**Sedative Hypnotics**	0.61	0.34	1.06
**Ethanol**	1.98	1.03	3.94
**Antimicrobials**	0.07	0.01	0.27
**MDMA Metab. & Analogs**	7.11	1.99	45.81
**Muscle Relaxants**	10.61	3.51	35.97
***Lithium**	NA	NA	NA
**Nicotine**	0.08	0.01	0.31

Supp. Table S3 Legend. Multivariate adjusted odds ratios for risk of death per additional drug added on from each class. 95% Confidence intervals reported. Adjusted Odds Ratios (aOR) greater than 1 indicate increased risk of death. Adjusted Odds Ratios less than 1 indicate decreased risk of death. (*Note that the aOR for Lithium is incalculable due to no occurrences of death in the study).

Table S4. Multivariate Adjusted Odds Ratios for Odds of Death, Binary Predictors

Class Name	aOR	95%CI-Lo	95%CI-Hi
**Opioids**	2.05	1.46	2.90
**Antidepressants**	1.62	1.11	2.36
**Benzodiazepines**	2.25	1.61	3.15
**Amphetamines & Stimulants**	3.05	2.13	4.41
**Cannabinoids**	0.33	0.22	0.48
**Antipsychotics**	0.40	0.25	0.62
**Cocaine**	0.42	0.28	0.62
**Dissociative Anesthetics**	1.42	0.83	2.45
**Psychedelics**	0.26	0.13	0.50
**Anesthetics**	7.13	2.37	26.93
**HIV**	0.10	0.01	0.38
**AEDs**	0.83	0.44	1.54
**Sedative Hypnotics**	0.78	0.43	1.40
**Ethanol**	2.03	1.06	3.99
**Antimicrobials**	0.06	0.01	0.27
**MDMA Metab. & Analogs**	8.71	2.46	55.49
**Muscle Relaxants**	8.17	2.87	25.98
***Lithium**	NA	NA	NA
**Nicotine**	0.09	0.01	0.35

Supp. Table S4 Legend. Multivariate adjusted odds ratios for risk of death associated with each drug class (coded as a binary predictor). 95% Confidence intervals reported. Adjusted Odds Ratios (aOR) greater than 1 indicate increased risk of death. Adjusted Odds Ratios less than 1 indicate decreased risk of death. (*Note that the aOR for Lithium is incalculable due to no occurrences of death in the study).

Table S5. Univariate Odds Ratios for Odds of Death, Antidepressants

Class Name	OR	95%CI-Lo	95%CI-Hi
**bupropion (n= 40)**	2.82	1.43	5.96
**paroxetine (n= 53)**	0.17	0.07	0.34
**fluoxetine (n= 62)**	0.20	0.10	0.38
**venlafaxine (n= 66)**	1.97	1.19	3.36
**citalopram (n= 56)**	1.92	1.10	3.42
**lofepramine (n= 30)**	0.11	0.03	0.31
**sertraline (n=26)**	2.36	1.05	5.80
**mirtazapine (n= 19)**	0.36	0.12	0.95

Supp. Table S5 Legend. Univariate unadjusted odds ratios for risk of death with each individual antidepressant. 95% Confidence intervals reported. Odds Ratios greater than 1 indicate increased risk of death. Odds Ratios less than 1 indicate decreased risk of death.

Table S6. Univariate Odds Ratios for Odds of Death, Antipsychotics

Class Name	OR	95%CI-Lo	95%CI-Hi
**olanzapine (n= 71)**	1.86	1.13	3.10
**aripiprazole (n= 33)**	0.07	0.01	0.22
**quetiapine (n= 54)**	0.64	0.41	0.97
***metoclopramide (n=18)**	NA	NA	NA
**clozapine (n= 11)**	0.10	0.01	0.53
***risperidone (n= 11)**	NA	NA	NA

Supp. Table S6 Legend. Univariate unadjusted odds ratios for risk of death with each individual antipsychotic. 95% Confidence intervals reported. Odds Ratios greater than 1 indicate increased risk of death. Odds Ratios less than 1 indicate decreased risk of death. (*Note that the odds ratio for metoclopramide is incalculable due to no occurrences of survival in the study, conversely the odds ratio for risperidone is incalculable due to no occurrences of death during the study).
